# Physical Fitness and Physical Function in Patients With Fabry Disease: A Cross‐Sectional Multicentre Study

**DOI:** 10.1002/jcsm.70233

**Published:** 2026-02-18

**Authors:** Nicola Vitturi, Giorgia Gugelmo, Andrea Gasperetti, Federica Duregon, Alessandro Dalmonico, Livia Lenzini, Sara Sponchiado, Gianni Carraro, Giacomo Marchi, Mattia Cominacini, Claudia Momentè, Federica Baciga, Claudia Baschirotto, Federica Caccia, Domenico Girelli, Andrea Ermolao, Gian Paolo Fadini, Yuri Battaglia

**Affiliations:** ^1^ Department of Medicine‐DIMED, Division of Metabolic Diseases University Hospital of Padova Padova Italy; ^2^ Sports and Exercise Medicine Division, Department of Medicine‐DIMED University Hospital of Padova Padova Italy; ^3^ Department of Medicine‐DIMED University Hospital of Padova Padova Italy; ^4^ Nephrology, Dialysis and Transplantation Unit, Department of Medicine University Hospital of Padova Padova Italy; ^5^ Department of Medicine, Section of Internal Medicine University of Verona, MetabERN Referral Center, Azienda Ospedaliera Universitaria Integrata Verona Italy; ^6^ Department of Engineering for Innovative Medicine University of Verona Verona Italy; ^7^ Department of Medicine University of Verona Verona Italy; ^8^ Nephrology and Dialysis Unit Pederzoli Hospital Peschiera del Garda Italy

**Keywords:** body composition, exercise, fatigue, physical activity, physical performance

## Abstract

**Background:**

Fabry disease (FD) is a rare, X‐linked lysosomal storage disorder affecting multiple organs, including the musculoskeletal system. The physical status of FD patients remains poorly characterized. This multicentre cross‐sectional study aimed to evaluate physical fitness and function in FD patients and investigate associations with sex, FD phenotype and treatment status.

**Methods:**

Adults (aged ≥ 18 years) with genetically confirmed FD were recruited. Demographic and laboratory data were collected. Physical fitness was assessed using cardiopulmonary exercise testing (VO_2_ peak) and body composition parameters (fat‐free mass index [FFMI], fat mass index [FM] and phase angle [PA]) via bioelectrical impedance analysis. Physical function was evaluated with performance tests (6‐min walk test, handgrip strength test, 30‐s chair‐stand test, short physical performance battery), muscle strength tests (isometric and isokinetic knee strength) and self‐report fatigue questionnaires. Statistical analyses were stratified by sex, phenotype (classic vs. late‐onset/Variants of Uncertain Significance [VUS]) and treatment status (enzyme replacement therapy [ERT]/chaperone‐treated versus untreated).

**Results:**

Forty‐two FD patients (13 males; mean age 46 ± 13.9 years) were enrolled. VO_2_ < 85% of predicted was more frequent in classic phenotype patients (53.8%) than in late‐onset/VUS (11.5%; *p* < 0.01). FFMI was lower in classic than late‐onset/VUS (16.8 ± 1.0 vs. 18.6 ± 2.1 kg/m^2^; *p* = 0.01). Treated males had lower PA than untreated males (4.8° ± 1.0° vs. 7.6° ± 0.9°; *p* = 0.04), and PA correlated with VO_2_ peak (*r* = 0.879; *p* = 0.01). Among classic phenotype males, 74.3% scored below the 50th percentile in handgrip strength (26.1 ± 7.8 kg), and 60.9% performed below predicted values in the 30‐s chair‐stand test (12.4 ± 4.3 repetitions). Self‐reported fatigue scores were higher in classic versus late‐onset/VUS patients (*p* = 0.05) and in treated patients compared to untreated patients (*p* = 0.02).

**Conclusions:**

Classic FD phenotype, particularly in males, was associated with reduced exercise capacity, muscle mass and physical performance. These findings support the integration of cardiopulmonary exercise testing, physical functional assessments and body composition analysis into the routine evaluation of FD patients.

## 1 Introduction

Fabry disease (FD) is a rare X‐linked lysosomal storage disorder caused by a deficiency of the enzyme α‐galactosidase A (α‐Gal A) [1]. The inactivity of this enzyme leads to the accumulation of globotriaosylsphingosine (GB3) in the lysosomes of various cell types, tissues and organs, resulting in progressive multi‐organ dysfunction [2].

However, the molecular pathology of FD is complex, involving not only GB3 accumulation but also mitochondrial and lysosomal dysfunction, endothelial dysfunction and abnormalities in autophagy [3]. These mechanisms contribute to the complexity of a genotype–phenotype correlation, which remains elusive [4].

Patients with FD exhibit a broad spectrum of signs and symptoms [5] that vary significantly based on sex, gene mutations and α‐Gal A activity levels [6]. Overall, two phenotypes of FD have been described: (a) the ‘classic’ phenotype, characterized by early onset, severe symptoms, markedly reduced or absent α‐Gal A activity and elevated serum GB3 (lysoGB3) levels [7]; (b) the ‘late onset’ phenotype, which typically manifests in adulthood, progresses more slowly, presents with milder symptoms and is linked to residual α‐Gal A activity [6]. Males with classic and late‐onset phenotypes typically exhibit a more severe phenotype than females due to X‐chromosome inactivation, also known as lyonization [8, 9].

Patients with the classic phenotype may also present with insidious, nonspecific symptoms frequently affecting the musculoskeletal system, such as fatigue and exercise intolerance [10]. Bierer et al. attributed the reduced exercise tolerance and fatigue of 39 FD patients to an average decline of 9 mmHg in diastolic pressure during exertion [11]. More recently, a pilot study showed that 14 FD patients reported improvements in well‐being, daily functioning and reduced fatigue after 12 months of strength/circuit exercise training [12].

In order to help prevent musculoskeletal symptoms [10, 12] and effectively reduce fatigue, specific treatments, such as enzyme replacement therapy (ERT) and oral chaperone [13], are currently available [10, 14]. However, despite these treatments, patients undergoing ERT may still experience limitations in physical activity and exercise intolerance [15, 16].

Among the valuable diagnostic tools for identifying physiological impairments and functional limitations in FD patients, cardiopulmonary exercise testing and physical function assessments have been proposed [17, 18, 19]; however, the available data are limited.

Therefore, this multicentre cross‐sectional study primarily aims to evaluate physical fitness, assessed by non‐invasive cardiopulmonary exercise test and bioimpedance analysis, as well as physical function, measured by physical performance tests, physical strength tests and self‐reported questionnaires on perceived fatigue, in patients with FD. Secondarily, any association of physical fitness and function with sex, FD phenotype and ERT/chaperone therapy was also examined.

## 2 Methods

### 2.1 Population

Ongoing outpatients were recruited between 1 December 2024 and 28 Feb 2025 from the Division of Metabolic Diseases of the University Hospital of Padova (Padova, Italy), the Nephrology Unit of Pederzoli Hospital, Peschiera del Garda (Verona, Italy) and the Division of Internal Medicine of the University Hospital of Verona (Verona, Italy).

Adult FD patients (> 18 years) with classic mutations, late‐onset mutations or Variants of Uncertain Significance (VUS) who exhibited clinical FD manifestations and/or were receiving therapy (ERT or chaperone) [6] were included.

Patients with physical disabilities (e.g., major lower limb amputation) or clinical limitations (e.g., severe effort angina, stage 4 NYHA heart failure or any intercurrent illness requiring hospitalization) that prevented assessment of physical function and fitness assessment were excluded.

Medical history (i.e., comorbidities and medications), physical examination (i.e., blood pressure, heart rate), anthropometric data (i.e., weight, height and body mass index [BMI]) and clinical parameters (i.e., sex, age, genetic analysis, FD phenotype and age at FD diagnosis) were recorded during routine multidisciplinary visits at each clinical centre (further details are provided in Tables 1 and S1).

**TABLE 1:** Characteristics of the Fabry disease patients (*n* = 42).

Variable	
Age, mean (SD) years	46.0 (13.9)
Sex, *n* (%)	Males, 13 (31)
	Females, 29 (69)
GLA mutations (*n*)	1077dupT (1)
	A143T (3)
	A73V (3)
	D313Y (1)
	E260K (1)
	G138E (1)
	G35R (1)
	G39fs (2)
	I91T (2)
	M290T (1)
	N215S (14)
	Q212Profs* (1)
	Q279K (2)
	R301X (2)
	S126G (2)
	W287* (4)
FD phenotype, *n* (%)	Classical, 13 (31.0)
	Late‐onset, 26 (61.9)
	VUS, 3 (7.1)
FD therapy, *n* (%)	ERT, 19 (45.2)
	Chaperonic, 7 (16.7)
	None, 16 (38.1)
Age at FD diagnosis, mean (SD) years	36.1 (19.7)
MET/day, mean (SD)	Males, 1950.8 (1779.2)
	Females, 1652.4 (2252.4)
Daily minutes of sedentary behaviour, mean (SD)	Males, 445.0 (212.1)
	Females, 430.8 (214.2)

Abbreviations: ERT, enzyme replacement therapy; MET, metabolic equivalent task; VUS, Variant of Uncertain Significance.

All data were handled in compliance with current regulations, including the EU Regulation 2016/679 (GDPR). Informed consent was obtained from all participants after providing them with adequate information about the study. This work was conducted according to the Helsinki Declaration 1975, revised in 2013, and approved by the Research Ethical Committee of the University Hospital of Padova (Ref. No. 6125/AO/24, AOP3572). However, it was not pre‐registered in any clinical trial registry.

### 2.2 Cardiorespiratory Fitness

Cardiorespiratory fitness was evaluated with a non‐invasive cardiopulmonary exercise test (CPET), using the Vyaire Medical GmbH, Vyntus CPX (Hoechberg, Germany), following the American Thoracic Society standards [20] (Table S1). CPET was performed using either a treadmill (Bruce Ramp or Modified Bruce Ramp) or a cycle ergometer (5 W × 1 to 15 W × 1 ramp protocol), depending on the patient's physical capacity. Maximal effort was defined by achieving at least one of the following criteria: a respiratory exchange ratio (RER) > 1.10, a Borg scale rating of perceived exertion > 18/20, a heart rate > 85% of age‐predicted maximum and/or a VO_2_ peak plateau [20].

CPET precisely defines the maximum exercise capacity by measuring peak oxygen uptake (VO_2_, mL/kg/min). A reduced VO_2_ peak was defined as < 85% of the predicted value and classified as mild (84%–75%), moderate (74%–50%) or severe (< 50%) reduction, respectively [21].

In patients with VO_2_ peak lower than 85% of the predicted value, further evaluation was performed, including (a) baseline electrocardiogram (ECG) to assess heart rhythm; (b) transthoracic echocardiogram (TTE) to evaluate heart structure and function; (c) pulmonary function testing (spirometry) to measure lung capacity. Based on CPET, resting echocardiography and complete blood count (CBC), patients with reduced VO_2_ on CPET were classified as (a) cardiac limitation (e.g., ejection fraction < 50%, chronotropic incompetence at CPET, reduction and/or early plateau in O_2_ pulse ventilation/carbon dioxide production [VE/VCO_2_] slope > 34, exercise‐induced ischaemic symptoms and/or ECG signs), (b) ventilatory limitation (e.g., breathing reserve < 15%, VE/VCO_2_ slope > 34, exercise‐induced desaturation) and (c) peripheral limitation (e.g., anaemia, no other cardiac and/or ventilatory limitation criteria).

### 2.3 Body Composition

Body composition was assessed by multi‐frequency bioelectrical impedance analysis (BIA) (InBody S10). Phase angle (PA), fat‐free mass index (FFMI) and the fat mass index (FMI) were estimated by BIA, and reference values from the general population were used for anthropometric measures and body composition parameters (Table S1). Two electrodes, placed at least 5 cm apart, were attached to the same side of the arm and leg of supine patients. Measurements were performed during a routine outpatient visit at the clinical centres. Patients were instructed to take their meals at least 2 h before BIA to reduce potential interference from food intake.

### 2.4 Physical Function

Physical function was assessed through the handgrip strength test (HG), the 6‐min walk test (6MWT) [22], the 30‐s chair stand test (30‐STS) [23] and the Short Physical Performance Battery (SPPB) [24] including gait speed (GS) test, sit‐and‐stand test and balance test [25] (Table S1).

Furthermore, the isokinetic machine assessed the isometric strength of thigh extensors and the isokinetic muscle strength of the knees (Table S1).

Briefly, the isometric strength test of the thigh extensors consisted of a maximum contraction of both thighs and was measured as maximum bilateral isometric knee extension torque per kilogram.

The isokinetic strength test of the knees was determined by comparing maximum flexion to maximum extension of the extensors and flexors of both thighs.

Physical function test results were compared to reference values from the general population (Table S1).

### 2.5 Questionnaire

Global Physical Activity Questionnaire (GPAQ), Bell CFIDS disability scale and Fatigue Impact Scale (FIS‐40) were administered to assess physical habits, functional ability and perceived fatigue, respectively (Table S1).

GPAQ assesses physical activity levels across work, travel and leisure time, measuring intensity and duration [26].

Bell CFIDS Disability Scale evaluates functional capacity and activity limitations in individuals with chronic fatigue, ranging from 0 (severe disability) to 100 (fully functional) [27].

FIS‐40 measures the impact of fatigue on physical, cognitive and psychosocial functioning using a 40‐item questionnaire [28]. The physical (FIS40F), cognitive (FIS40C) and psychosocial (FIS40P) subscores provide a measure of the impact of fatigue on physical activities, mental processes and social and emotional consequences, respectively.

### 2.6 Statistical Analysis

Statistical analysis was performed using IBM SPSS Statistics version 29. Continuous variables were analysed for normality using histograms and QQ plots. Normality was further assessed using the Shapiro–Wilk test or the Kolmogorov–Smirnov test, and data were reported as the mean and standard deviation. Categorical variables were presented as absolute values and percentages. Correlations between continuous variables were assessed using Pearson and Spearman tests. Associations between categorical variables were evaluated using the chi‐squared and Fisher's exact tests. Student's *t*‐test and ANOVA were used to compare the clinical, physical and anthropometric parameters among patient subgroups based on sex, FD phenotype and therapy status. However, sex‐dependent variables, such as VO_2_ peak, HG, 30‐STS, isometric unilateral thigh torque max extension, isokinetic unilateral knee torque max extension and flexion, FFMI, FMI and PA, were analysed and compared within each sex subgroup to avoid sex‐related bias. No cross‐sex comparisons were performed for these variables (Table S1). A *p*‐value of less than 0.05 was considered statistically significant.

## 3 Results

### 3.1 Population

Among 50 FD patients screened (35 from the University Hospital of Padova, 10 from the University Hospital of Verona and 5 from Pederzoli Hospital), 42 were enrolled, while eight were excluded for refusal of consent. Demographic and clinical characteristics of the entire cohort are summarized in Table 1.

Overall, 69% (*n* = 29) of patients were female. The mean age was 46.0 years (SD 13.9), and the mean age at the time of FD diagnosis was 36.1 years (SD 19.7). The most common FD phenotype was late‐onset (*n* = 26), followed by the classic (*n* = 13) and VUS (*n* = 3) [29].

Patients with late‐onset/VUS phenotype were significantly older (*p* = 0.02) and received an FD diagnosis at a later age (*p* = 0.02) than those with classic phenotype. There was no statistically significant difference in age at FD diagnosis between the treated (36.6 years [SD 18.5]) and the untreated group (35.1 years [SD 22.2]).

ERT and chaperone therapy were administered to 19 and 7 patients, respectively. Among treated patients, 12/26 had a classic phenotype (8 females, 4 males), while 14/16 untreated individuals had a late‐onset/VUS phenotype (11 females, 3 males). Notably, all patients with the classic phenotype were receiving ERT.

### 3.2 Cardiopulmonary Exercise Test

**FIGURE 1:**
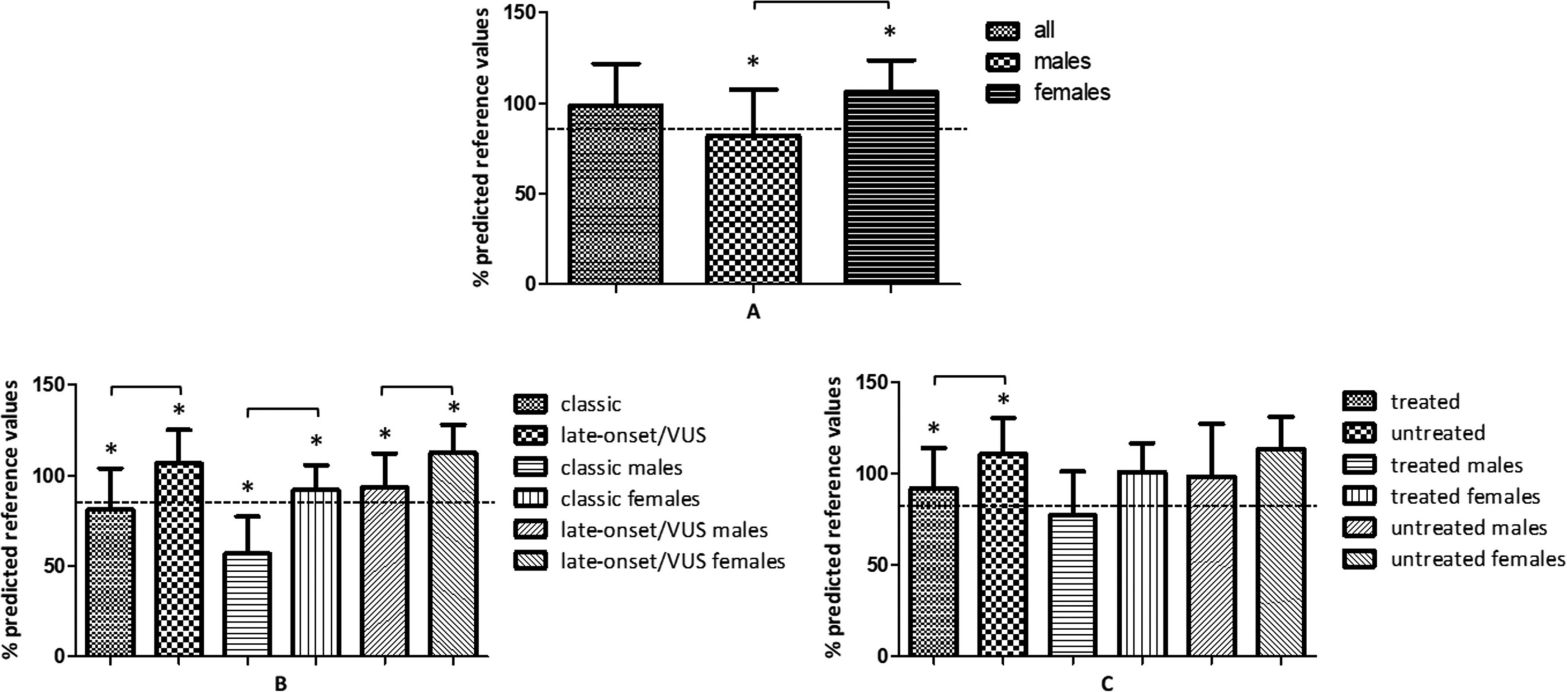
VO_2_ peak predicted percentage values in (A) the entire cohort and stratified by sex (42 total FD; 13 males, 29 females); (B) FD phenotypes and sex (13 classic, 29 late‐onset/VUS; 4 classic males, 9 late‐onset/VUS males; 9 classic females, 20 late‐onset/VUS females); (C) untreated/treated and sex (26 treated, 16 untreated; 10 treated males, 3 untreated males; 16 treated females, 3 untreated females). The dashed line represents the 85% reference threshold. All variables are normally distributed and are presented as mean ± standard deviation (SD).

CPET parameters are reported in Table 2. Notably, no statistically significant difference in VO_2_ peak between males (28.6 mL/kg/min [SD 13.4]) and females (26.7 mL/kg/min [SD 5.4]) was found (*p* = 0.63). A reduced percentage of the maximal VO_2_ predicted value was observed in 10 patients (23.8%), with five cases due to cardiac limitation and five cases due to peripheral limitation. Among these 10 patients, two had a severe reduction in VO_2_, three presented with a moderate VO_2_ impairment and five had a mild reduction in VO_2_. The percentage VO_2_ peak values relative to the predicted values across groups stratified by sex, FD phenotype and ERT/chaperone treatment are shown in Figure 1.

**TABLE 2:** Clinical characteristics and CPET parameters in males, females and the total cohort (*N* = 42) stratified by phenotype and ERT/chaperon treatment.

Variables	All sample (*n*)		FD phenotype (*n*)					ERT/chaperon therapy (*n*)				
			Classic		Late‐onset/VUS			Treated		Untreated		
Age, years	Total (42)	46.0 ± 13.9	Total (13)	39.1 ± 11.5	Total (29)	49.1 ± 13.9	***p* = 0.022** *	Total (26)	47.4 ± 13.3	Total (16)	43.8 ± 15	*p* = 0.433
	Males (13)	47.2 ± 19.1	Males (4)	37.5 ± 14.9	Males (9)	51.4 ± 19.9	*p* = 0.200	Males (10)	51.4 ± 16.4	Males (3)	33.0 ± 24.3	*p* = 0.319
	Females (29)	45.5 ± 11.2	Females (9)	39.8 ± 10.8	Females (20)	48.1 ± 10.7	*p* = 0.073	Females (16)	44.9 ± 10.7	Females (13)	46.2 ± 12.2	*p* = 0.756
		*p* = 0.724		*p* = 0.795		*p* = 0.641			*p* = 0.282		*p* = 0.447	
Age a diagnosis, years	Total (42)	36.1 ± 19.7	Total (13)	25.6 ± 17.2	Total (29)	40.8 ± 19.1	***p* = 0.018** *	Total (26)	36.7 ± 18.9	Total (16)	35.1 ± 21.4	*p* = 0.804
	Males (13)	36.5 ± 23.9	Males (4)	27.8 ± 17.2	Males (9)	40.4 ± 26.3	*p* = 0.329	Males (10)	41.6 ± 19.6	Males (3)	19.7 ± 34.1	*p* = 0.382
	Females (29)	35.9 ± 17.9	Females (9)	24.6 ± 181	Females (20)	41.0 ± 15.7	***p* = 0.035** *	Females (16)	33.6 ± 18.4	Females (13)	38.6 ± 17.6	*p* = 0.463
		*p* = 0.928		*p* = 0.771		*p* = 0.949			*p* = 0.314		*p* = 0.439	
VO_2_ peak, mL/kg/min	Total (42)	27.3 ± 8.5	Total (13)		Total (29)			Total (26)		Total (16)		
	Males (13)	28.6 ± 13.4	Males (4)	24.8 ± 12.28	Males (9)	30.3 ± 14.2	*p* = 0.502	Males (10)	24.82 ± 9.62	Males (3)	41.2 ± 18.6	*p* = 0.262
	Females (29)	26.7 ± 5.4	Females (9)	28.7 ± 5.9	Females (20)	25.9 ± 5.0	*p* = 0.234	Females (16)	26.5 ± 5.7	Females (13)	27.0 ± 5.1	*p* = 0.830
		*p* = NA		*p* = NA		*p* = NA			*p* = NA		*p* = NA	
VO_2_ peak, % of patients with < 85% of the predicted value	Total (42)	23.8	Total (13)	53.8 (7)	Total (29)	10.3 (3)	***p* = 0.005** *	Total (26)	34.6 (9)	Total (16)	6.3 (1)	***p* = 0.036** *
	Males (13)	53.8 (7)	Males (4)	100 (4)	Males (9)	33.3 (3)	*p* = 0.07	Males (10)	60.0 (6)	Males (3)	33.3 (1)	*p* = 0.559
	Females (29)	10.3 (3)	Females (9)	33.3 (3)	Females (20)	0.0 (0)	***p* = 0.023** *	Females (16)	18.9 (3)	Females (13)	0.0 (0)	*p* = 0.232
		***p* = 0.005** *		*p* = 0.070		***p* = 0.023** *			***p* = 0.046** *		*p* = 0.188	
VO_2_ peak, % predicted reference values	Total (42)	98.8 ± 23.0	Total (13)	81.2 ± 22.8	Total (29)	106.7 ± 18.6	***p* = 0.002** *	Total (26)	91.7 ± 22.1	Total (16)	110.4 ± 20.0	***p* = 0.008** *
	Males (13)	82.1 ± 25.5	Males (4)	56.75 ± 20.6	Males (9)	93.3 ± 18.8	***p* = 0.026** *	Males (10)	77.2 ± 23.9	Males (3)	98.3 ± 28.7	*p* = 0.333
	Females (29)	106.3 ± 17.5	Females (9)	92.1 ± 13.6	Females (20)	112.7 ± 15.4	***p* = 0.002** *	Females (16)	100.7 ± 15.7	Females (13)	113.2 ± 17.8	***p* = 0.060** *
		***p* = 0.006** *		***p* = 0.032** *		***p* = 0.018** *			***p* = 0.015** *		*p* = 0.469	
OUES	Total (42)	1965.5 ± 533.7	Total (13)	1776.2 ± 459.5	Total (29)	2037.3 ± 549.4	*p* = 0.144	Total (26)	1956.7 ± 484.4	Total (16)	1978.8 ± 616.7	*p* = 0.905
	Males (13)	2159 ± 831	Males (4)	1562.3 ± 793.5	Males (9)	2357 ± 783.8	*p* = 0.160	Males (10)	2075.8 ± 711.6	Males (3)	2408.7 ± 1284.2	*p* = 0.704
	Females (29)	1882.6 ± 325.8	Females (9)	1856.4 ± 307.8	Females (20)	1893.1 ± 339.2	*p* = 0.786	Females (16)	1885.2 ± 285.7	Females (13)	1879.5 ± 379.0	*p* = 0.965
		*p* = 0.135		*p* = 0.372		***p* = 0.032** *			*p* = 0.362		*p* = 0.095	
RER max	Total (42)	1.17 ± 0.09	Total (13)	1.14 ± 0.09	Total (29)	1.18 ± 0.10	*p* = 0.234	Total (26)	1.16 ± 0.10	Total (16)	1.19 ± 0.08	*p* = 0.218
	Males (13)	1.19 ± 0.09	Males (4)	1.19 ± 0.09	Males (9)	1.18 ± 0.10	*p* = 0.922	Males (10)	1.20 ± 0.10	Males (3)	1.14 ± 0.04	*p* = 0.195
	Females (29)	1.16 ± 0.10	Females (9)	1.12 ± 0.09	Females (20)	1.18 ± 0.10	*p* = 0.139	Females (16)	1.13 ± 0.09	Females (13)	1.20 ± 0.09	***p* = 0.034**
		*p* = 0.212		*p* = 0.124		*p* = 0.881			*p* = 0.085		*p* = 0.280	

*Note:* Data are expressed as mean ± standard deviation.

Abbreviations: CPET, cardiopulmonary exercise test; NA, not applicable; OUES, oxygen uptake efficiency slope; RER, respiratory exchange ratio; SD, standard deviation; VO_2_, volume of oxygen consumption; VUS, Variant of Uncertain Significance.

* Statistically significant.

*Regarding sex*, a statistically significant association was found between sex and a reduction in VO_2_ peak (*p* = 0.006). VO_2_ peak predicted percentage values in males (82.1% [SD 25.5]) were significantly lower than in females (106.3% [SD 17.5]) (*p* = 0.006). Similarly, males with VO_2_ peak levels under 85% of the expected value (53.8%, 7/13) were more frequent than females (10.3%, 3/26) (*p* = 0.005).

*Regarding phenotype*, patients with the classic phenotype were more likely to have VO_2_ predicted < 85% (53.8%, 7/13) compared to those with the late‐onset/VUS phenotype (10.3%, 3/29) (*p* = 0.005), including females (*p* = 0.023). Furthermore, VO_2_ peak predicted percentage values in the classic group (81.2% [SD 22.8]) were significantly lower than in the late‐onset/VUS group (106.7% [SD 18.6]) (*p =* 0.002), even when stratified by sex (females [*p* = 0.002] and males [*p* = 0.026]).

*Regarding therapy status*, patients undergoing ERT/chaperone were more likely to have VO_2_ peak values below 85% of the predicted maximum (34.6%, 8/21) compared to untreated patients (6.3%, 1/16) (*p =* 0.036). VO_2_ peak predicted percentage values in treated individuals (91.7% [SD 22.1]) were significantly lower than in untreated ones (110.4% [SD 20.0]) (*p =* 0.008).

### 3.3 Anthropometrics and Body Composition

#### 3.3.1 Body Mass Index

Overall, average BMI was 25.3 (SD 6.5) kg/m^2^, with no statistically significant differences observed between males (25.6 kg/m^2^ [SD 6.2]) and females (25.2 kg/m^2^ [SD 6.8]) or treated (24.7 kg/m^2^ [SD 6.0]) and untreated (26.2 kg/m^2^ [SD 6.4]) patients (Table 3). However, a significantly lower BMI was found in patients with classic phenotype (20.7 kg/m^2^ [SD 1.8]) compared to those with late‐onset/VUS phenotype (27.6 kg/m^2^ [SD 6.7]) (*p* = 0.001), in both males (*p* = 0.002) and females (*p* = 0.030).

**TABLE 3:** Anthropometrics and body composition parameters in males, females and the total cohort (*N* = 35) stratified by phenotype and ERT/chaperon treatment.

Variables	All sample (*n*)		FD phenotype (*n*)					ERT/chaperon therapy (*n*)				
			Classic		Late‐onset/VUS			Treated		Untreated		
BMI, kg/m^2^	Total (42)	25.3 ± 6.5	Total (13)	20.7 ± 1.8	Total (29)	27.3 ± 6.8	***p* = 0.001** *	Total (26)	24.7 ± 6.0	Total (16)	26.2 ± 7.4	*p* = 0.485
	Males (13)	25.6 ± 6.2	Males (4)	19.7 ± 1.6	Males (9)	28.2 ± 5.6	***p* = 0.002** *	Males (10)	25.9 ± 7.12	Males (3)	24.7 ± 0.6	*p* = 0.398
	Females (29)	25.2 ± 6.8	Females (9)	21.2 ± 1.9	Females (20)	27.0 ± 7.4	***p* = 0.030** *	Females (16)	24.1 ± 5.3	Females (13)	26.5 ± 8.2	*p* = 0.355
		*p* = 0.423		*p* = 0.193		*p* = 0.630			*p* = 0.502		*p* = 0.445	
FFMI, kg/m^2^	Total (35)		Total (9)		Total (26)			Total (20)		Total (15)		
	Males (10)	19.6 ± 3.1	Males (4)	16.7 ± 0.7	Males (6)	21.5 ± 2.4	***p* = 0.003** *	Males (8)	18.8 ± 2.9	Males (2)	22.7 ± 1.8	*p* = 0.058
	Females (25)	17.1 ± 1.8	Females (5)	16.0 ± 1.2	Females (20)	17.4 ± 1.8	***p* = 0.032** *	Females (12)	16.7 ± 1.4	Females (13)	17.5 ± 2.0	*p* = 0.278
		*p* = NA		*p* = NA		*p* = NA			*p* = NA		*p* = NA	
FMI, kg/m^2^	Total (35)		Total (9)		Total (26)			Total (20)		Total (15)		
	Males (10)	5.2 ± 4.1	Males (4)	3.1 ± 1.0	Males (6)	6.6 ± 4.8	*p* = 0.099	Males (8)	6.1 ± 4.1	Males (2)	1.7 ± 1.3	*p* = 0.093
	Females (25)	8.3 ± 5.2	Females (5)	4.6 ± 1.5	Females (20)	9.3 ± 5.4	***p* = 0.003** *	Females (12)	8.0 ± 4.9	Females (13)	8.6 ± 5.6	*p* = 0.790
		*p* = NA		*p* = NA		*p* = NA			*p* = NA		*p* = NA	
PA, grade	Total (35)		Total (9)		Total (26)			Total (20)		Total (15)		
	Males (10)	5.4 ± 1.5	Males (4)	4.8 ± 1.0	Males (6)	5.8 ± 1.7	*p* = 0.125	Males (8)	4.8 ± 1.0	Males (2)	7.6 ± 0.9	***p* = 0.036** *
	Females (25)	5.6 ± 0.7	Females (5)	5.8 ± 0.8	Females (20)	5.5 ± 0.7	*p* = 0.496	Females (12)	5.5 ± 0.6	Females (13)	5.6 ± 0.8	*p* = 0.780
		*p* = NA		*p* = NA		*p* = NA			*p* = NA		*p* = NA	

*Note:* Data are expressed as mean ± standard deviation.

Abbreviations: BMI, body mass index; FFMI, free fat mass index; FMI, fat mass index; NA, not applicable; PA, phase angle; SD, standard deviation; VUS, Variant of Uncertain Significance.

* Statistically significant.

#### 3.3.2 Fat‐Free Mass Index and Fat Mass Index

**FIGURE 2:**
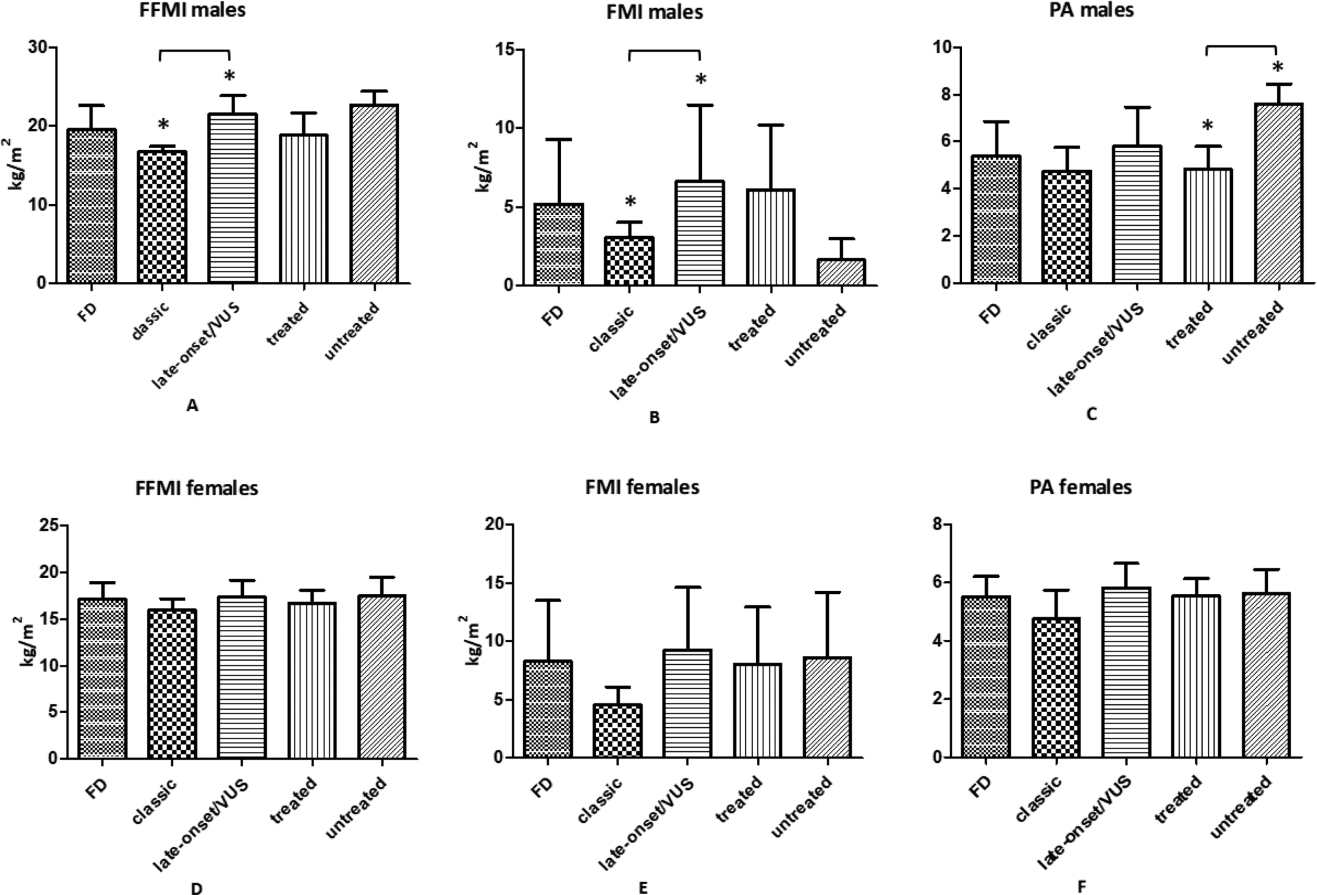
FFMI, FMI and PA in (A, B, C) 10 males (4 classic, 6 late‐onset/VUS; 8 treated, 2 untreated) and (D, E, F) 25 females (5 classic, 20 late‐onset/VUS; 12 treated, 13 untreated). All variables are normally distributed and are presented as mean ± standard deviation (SD).

*Regarding males*, average FFMI was 19.6 kg/m^2^ (SD 3.1) in the reference range of 18.7–21.0 kg/m^2^ (Figure 2A). Patients with the classic phenotype had significantly lower FFMI (16.7 kg/m^2^ [SD 0.7]) compared to those with the late‐onset/VUS phenotype (21.5 kg/m^2^ [SD 2.4]) (*p* = 0.003) (Figure 2A). Additionally, FFMI was significantly higher in males with VO_2_ peak values below 85% of the predicted maximum than in males with normal VO_2_ peak values (*p* = 0.038).

FMI (reference range 4.2–7.0 kg/m^2^) was 3.1 (SD 1.0) kg/m^2^ and 6.6 kg/m^2^ (SD 4.9) in the classic and late‐onset/VUS phenotype, respectively (Figure 2B). No statistically significant differences were found between or within groups.

*Regarding females*, FFMI (reference range 14.9–17.2 kg/m^2^) was significantly lower in classic phenotype (16.0 kg/m^2^ [SD 1.2]) than in late‐onset/VUS phenotype (17.4 kg/m^2^ [SD 1.8]) (*p* = 0.032) (Figure 2D). Furthermore, FFMI was significantly higher in females with VO_2_ peak values over 85% of the predicted maximum than in females with reduced VO_2_ peak values (*p* = 0.050).

FMI (reference range 6.9–10.6 kg/m^2^) was significantly lower in classic phenotype (4.6 kg/m^2^ [SD 1.5]) compared to late‐onset/VUS phenotype (9.3 kg/m^2^ [SD 5.4]) (*p* = 0.003) (Figure 2E). FMI was significantly higher in individuals with VO_2_ peak values under 85% of the predicted maximum compared to females with normal VO_2_ peak values (*p* = 0.050). Moreover, higher FMI was inversely associated with lower VO_2_ peak (mL/kg/min) (*r* = −0.591, *p* = 0.002).

In both sexes, treated or untreated patients did not show significant differences in FFMI and FMI.

#### 3.3.3 Phase Angle

*Regarding males*, average PA was 5.4° (SD 1.5) (Figure 2C) below the reference range of 6.14°–7.59°. No statistically significant differences among phenotype groups were found (*p* = 0.125). However, PA was significantly lower in treated patients (*p* = 0.036) than in untreated patients. Higher PA scores significantly correlated with greater VO_2_ peak predicted percentage values (*r* = 0.879, *p* = 0.01) and resulted in higher values in subjects with VO_2_ peak values exceeding 85% of the predicted maximum (*p* = 0.04).

*Regarding females*, average PA was 5.6° (SD 0.7) (reference range 5.39°–6.74°) (Figure 2F). No significant differences for phenotype, therapy status or predicted VO_2_ were found.

### 3.4 Physical Function

#### 3.4.1 Six‐Minute Walking Test and Short Physical Performance Battery

Analysing the entire cohort, 6MWT distance ranged from 240 to 702 m, with a mean value of 529.4 m (SD 95.4). SPPB Total Score had a mean of 11.1 (SD 1.3), ranging from 7 to 12, with 8.1% of the patients showing an SPPB score ≤ 8. No statistically significant differences were found between groups stratified by sex, FD phenotype, and treatment in performance test scores (Table 4). However, 6MWT, predicted 6MWT distance and SPPB Total Score tended to be lower in males with classic phenotype compared to other groups.

**TABLE 4:** Performance parameters in males, females and the total cohort (*N* = 35) stratified by phenotype and ERT/chaperon treatment.

Variables	All sample (*n*)		FD phenotype (*n*)					ERT/chaperon therapy (*n*)				
			Classic		Late‐onset/VUS			Treated		Untreated		
6MWT, meters	Total (35)	529.4 ± 95.4	Total (7)	520.0 ± 129.9	Total (28)	529.4 ± 95.4	*p* = 0.826	Total (19)	516.7 ± 97.9	Total (16)	544.6 ± 93.2	*p* = 0.397
	Males (11)	515.4 ± 135.7	Males (3)	455.3 ± 190.5	Males (8)	537.9 ± 117.5	*p* = 0.540	Males (8)	487.8 ± 126.8	Males (3)	589.0 ± 156.8	*p* = 0389
	Females (24)	535.9 ± 72.9	Females (4)	568.5 ± 46.9	Females (20)	529.4 ± 76.3	*p* = 0.221	Females (11)	537.7 ± 69.5	Females (13)	534.3 ± 78.4	*p* = 0.911
		*p* = 0.563		*p* = 0.146		*p* = 0.853			*p* = 0.336		*p* = 0.611	
6MWT, % predicted reference values	Total (35)	91.7 ± 15.9	Total (7)	87.0 ± 22.3	Total (28)	91.7 ± 15.9	*p* = 0.262	Total (19)	90.0 ± 17.4	Total (16)	93.8 ± 14.1	*p* = 0.482
	Males (11)	87.8 ± 22.2	Males (3)	75.3 ± 31.5	Males (9)	92.4 ± 18.2	*p* = 0.451	Males (8)	85.4 ± 23.4	Males (3)	94.2 ± 21.5	*p* = 0.588
	Females (24)	93.5 ± 12.1	Females (4)	95.7 ± 9.8	Females (20)	93.1 ± 12.7	*p* = 0.658	Females (11)	93.3 ± 11.5	Females (13)	93.7 ± 13.1	*p* = 0.944
		*p* = 0.328		*p* = 0.266		*p* = 0.465			*p* = 0.399		*p* = 0.975	
SPPB, total Score	Total (35)	11.1 ± 1.3	Total (7)	11.3 ± 1.5	Total (28)	11.1 ± 1.3	*p* = 0.358	Total (19)	10.8 ± 1.6	Total (16)	11.5 ± 0.7	*p* = 0.182
	Males (11)	10.9 ± 1.5	Males (3)	10.7 ± 2.3	Males (9)	11.0 ± 1.2	*p* = 0.830	Males (8)	10.8 ± 1.6	Males (3)	11.3 ± 1.2	*p* = 0.532
	Females (24)	11.2 ± 1.3	Females (4)	11.8 ± 0.5	Females (20)	11.1 ± 1.4	*p* = 0.136	Females (11)	10.9 ± 1.8	Females (13)	11.5 ± 0.7	*p* = 0.315
		*p* = 0.278		*p* = 0.391		*p* = 0.423			*p* = 0.839		*p* = 0.830	

*Note:* Data are expressed as mean ± standard deviation.

Abbreviations: 6MWT, 6‐min walk test; SD, standard deviation; SPPB, Short Physical Performance Battery; VUS, Variant of Uncertain Significance.

#### 3.4.2 Handgrip Strength Test

**FIGURE 3:**
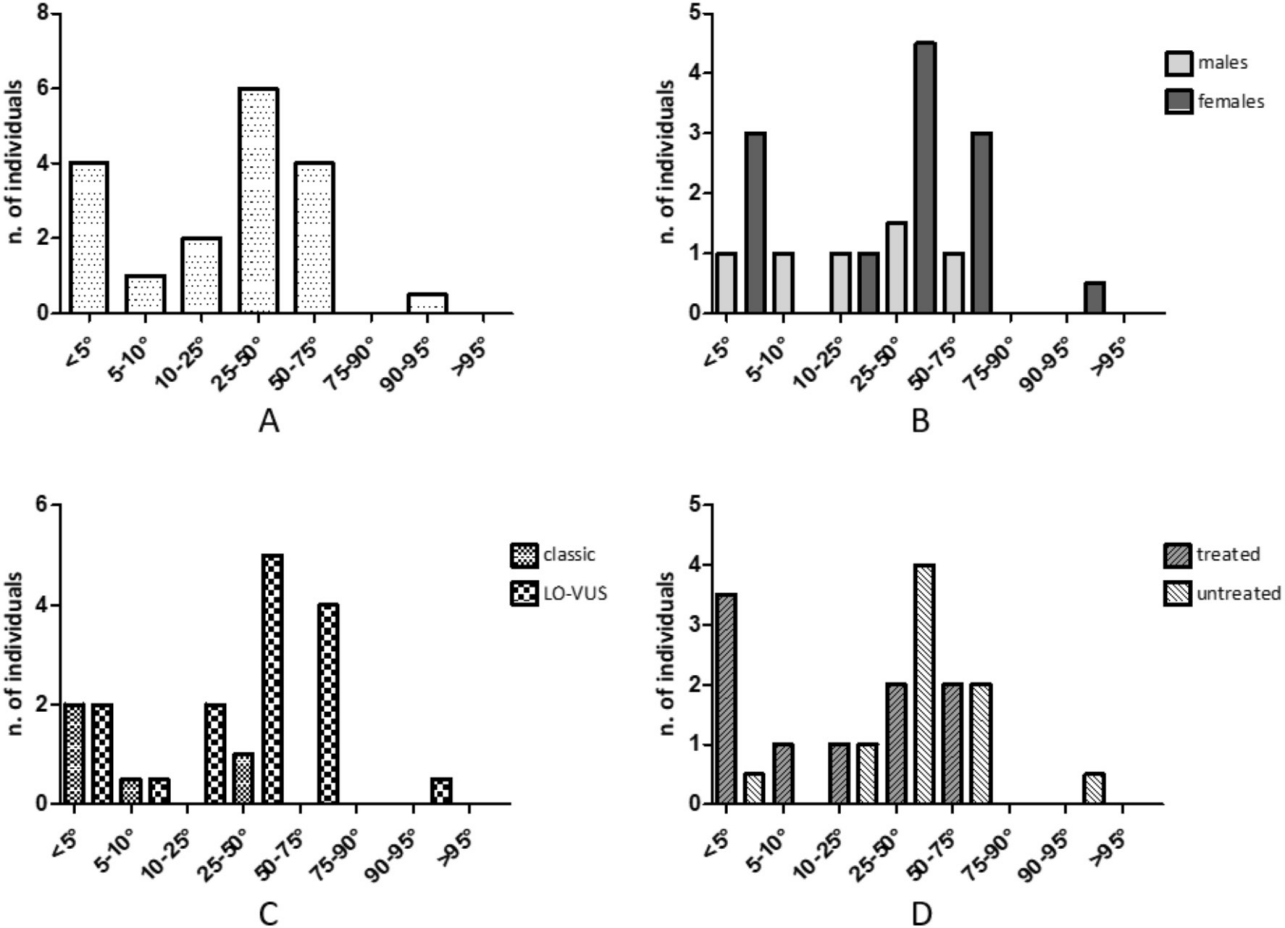
Handgrip percentiles in (A) the entire cohort (35 total FD); (B) different sex (10 males, 25 females); (C) phenotype subgroups (7 classic, 28 late‐onset/VUS); (D) treatment status (19 treated, 16 untreated).

HG test showed an average value for both hands of 37.6 kg (SD 4.7) in males and 24.5 kg (SD 5.6) in females, corresponding to 74.3% of individuals (81.8% of males and 70.8% of females) < 50th percentile by the HG test (Figure 3A,B) according to Canadian reference values [30] (Table 5).

**TABLE 5:** Strength parameters in males, females and the total cohort (*N* = 35) stratified by phenotype and ERT/chaperon treatment.

Variables	All sample (*n*)		FD phenotype (*n*)					ERT/chaperon therapy (*n*)				
			Classic		Late‐onset/VUS			Treated		Untreated		
Handgrip, kg	Total (35)		Total (7)		Total (28)			Total (19)		Total (16)		
	Males (11)	37.6 ± 4.7	Males (3)	33.4 ± 1.9	Males (8)	39.2 ± 4.4	***p* = 0.015** *	Males (8)	37.0 ± 4.8	Males (3)	39.2 ± 4.9	*p* = 0.537
	Females (24)	24.5 ± 5.6	Females (4)	21.9 ± 4.7	Females (20)	25.0 ± 5.7	*p* = 0.295	Females (11)	22.0 ± 6.22	Females (13)	26.6 ± 4.1	***p* = 0.043**
		*p* = NA		*p* = NA		*p* = NA			*p* = NA		*p* = NA	
Handgrip, *n* < 50° percentile	Total (35)	26	Total (7)	7	Total (28)	19	*p* = 0.153	Total (19)	11	Total (16)	15	*p* = 0.381
	Males (11)	9	Males (3)	3	Males (8)	6	*p* = 1.00	Males (8)	6	Males (3)	3	*p* = 1.00
	Females (24)	17	Females (4)	4	Females (20)	13	*p* = 0.283	Females (11)	9	Females (13)	8	*p* = 0.386
		*p* = 0.685		*p* = 0.000		*p* = 1.00			*p* = 1.00		*p* = 0.509	
30‐s chair stand, repetitions	Total (35)		Total (7)		Total (28)			Total (19)		Total (16)		
	Males (11)	13.2 ± 3.9	Males (3)	12.5 ± 6.5	Males (8)	13.5 ± 3.1	*p* = 0.819	Males (8)	12.9 ± 3.9	Males (3)	14.0 ± 4.6	*p* = 0.744
	Females (24)	13.1 ± 3.4	Females (4)	14.8 ± 3.0	Females (20)	12.7 ± 3.4	*p* = 0.289	Females (11)	12.2 ± 4.0	Females (13)	13.9 ± 2.7	*p* = 0.244
		*p* = NA		*p* = NA		*p* = NA			*p* = NA		*p* = NA	
30‐s chair stand, % predicted reference values	Total (35)	60.9 ± 15.8	Total (7)	58.7 ± 19.1	Total (28)	61.6 ± 15.2	*p* = 0.720	Total (19)	58.0 ± 17.5	Total (16)	64.7 ± 13.1	*p* = 0.222
	Males (11)	59.6 ± 17.5	Males (3)	49.9 ± 25.0	Males (8)	63.3 ± 14.2	*p* = 0.457	Males (8)	59.6 ± 20.0	Males (3)	59.7 ± 11.0	*p* = 0.993
	Females (24)	61.6 ± 15.3	Females (4)	65.3 ± 13.2	Females (20)	60.8 ± 16.0	*p* = 0.582	Females (11)	56.7 ± 16.1	Females (13)	66.1 ± 13.7	*p* = 0.170
		*p* = 0.750		*p* = 0.408		*p* = 0.701			*p* = 0.747		*p* = 0.446	
Isometric unilateral tights torque max extension corrected LL	Total (35)		Total (7)		Total (28)			Total (19)		Total (16)		
	Males (11)	2.3 ± 1.2	Males (3)	2.7 ± 1.1	Males (8)	2.2 ± 1.2	*p* = 0.277	Males (8)	2.4 ± 0.7	Males (3)	2.2 ± 2.2	*p* = 0.899
	Females (24)	1.9 ± 0.8	Females (4)	1.8 ± 0.95	Females (20)	1.9 ± 0.8	*p* = 0.735	Females (11)	1.8 ± 0.7	Females (13)	2.0 ± 0.8	*p* = 0.462
		*p* = NA		*p* = NA		*p* = NA			*p* = NA		*p* = NA	
Isometric unilateral tights torque max extension lower than LL reference values, % of population	Total (34)	61.8	Total (7)	71.4	Total (27)	55.2	*p* = 0.682	Total (19)	68.4	Total (15)	53.3	*p* = 0.484
	Males (11)	60.0	Males (3)	66.6	Males (8)	57.2	*p* = 1.00	Males (8)	62.5	Males (3)	50.0	*p* = 1.00
	Females (23)	62.5	Females (4)	75.0	Females (19)	60.0	*p* = 1.00	Females (11)	72.7	Females (12)	53.8	*p* = 0.423
		*p* = 1.00		*p* = 1.00		*p* = 1.00			*p* = 1.00		*p* = 1.00	
Isokinetic unilateral knee torque max extension	Total (35)		Total (7)		Total (28)			Total (19)		Total (16)		
	Males (11)	118.6 ± 38.5	Males (3)	102.3 ± 22.1	Males (8)	124.7 ± 42.7	*p* = 0.293	Males (8)	118.3 ± 32.2	Males (3)	119.2 ± 61.5	*p* = 0.984
	Females (24)	88.4 ± 34.2	Females (4)	83.3 ± 28.3	Females (20)	89.4 ± 35.8	*p* = 0.723	Females (11)	80.5 ± 32.8	Females (13)	95.2 ± 35.1	*p* = 0.301
		*p* = NA		*p* = NA		*p* = NA			*p* = NA		*p* = NA	
Isokinetic unilateral knee torque max extension, % predicted reference values	Total (35)	67.3 ± 23.2	Total (7)	68.4 ± 19.0	Total (28)	67.0 ± 24.4	*p* = 0.867	Total (19)	64.7 ± 23.0	Total (16)	70.3 ± 23.8	*p* = 0.493
	Males (11)	64.8 ± 16.4	Males (3)	61.3 ± 3.2	Males (8)	66.1 ± 19.3	*p* = 0.518	Males (8)	67.3 ± 13.7	Males (3)	58.3 ± 24.4	*p* = 0.600
	Females (24)	68.4 ± 25.9	Females (4)	73.5 ± 25.0	Females (20)	67.3 ± 26.6	*p* = 0.663	Females (11)	62.9 ± 28.4	Females (13)	73.0 ± 23.7	*p* = 0.362
		*p* = 0.680		*p* = 0.395		*p* = 0.898			*p* = 0.696		*p* = 0.449	
Isokinetic unilateral knee torque max flexion	Total (35)		Total (7)		Total (28)			Total (19)		Total (16)		
	Males (11)	44.3 ± 22.6	Males (3)	63.5 ± 15.3	Males (8)	60.2 ± 24.8	*p* = 0.797	Males (8)	59.8 ± 18.7	Males (3)	64.6 ± 33.	*p* = 0.833
	Females (24)	61.1 ± 21.9	Females (4)	50.2 ± 20.4	Females (20)	43.2 ± 23.3	*p* = 0.569	Females (11)	42.2 ± 21.5	Females (13)	46.1 ± 24.1	*p* = 0.676
		*p* = NA		*p* = NA		*p* = NA			*p* = NA		*p* = NA	
Isokinetic unilateral knee torque max flexion, % predicted reference values	Total (35)	75.6 ± 34.0	Total (7)	90.7 ± 34.8	Total (28)	71.7 ± 33.3	*p* = 0.190	Total (19)	75.4 ± 32.5	Total (16)	75.8 ± 36.7	*p* = 0.972
	Males (11)	65.6 ± 21.2	Males (3)	75.7 ± 17.2	Males (8)	61.9 ± 22.3	*p* = 0.164	Males (8)	66.4 ± 19.6	Males (3)	63.7 ± 29.7	*p* = 0.894
	Females (24)	80.4 ± 38.1	Females (4)	102 ± 42.7	Females (20)	75.8 ± 36.7	*p* = 0.317	Females (11)	82.6 ± 39.5	Females (13)	78.6 ± 38.6	*p* = 0.811
		*p* = 0.243		*p* = 0.324		*p* = 0.240			*p* = 0276		*p* = 0.503	

*Note:* Data are expressed as mean ± standard deviation.

Abbreviations: LL, lower limit; NA, not applicable; SD, standard deviation; VUS, Variant of Uncertain Significance.

* Statistically significant.

When comparing *sex and therapy groups*, a statistically significant difference in HG between treated and untreated females was found (*p* = 0.043).

*Regarding phenotype*, classic males presented significantly lower HG scores than late‐onset/VUS males (*p* = 0.015). Indeed, all classic form patients were below the 50th percentile, while 65.5% of late‐onset/VUS patients were below the 50th percentile. Moreover, the classic group showed more HG scores in the lowest percentiles (< 25th percentile) (Figure 3C).

*Regarding therapy status*, a high percentage of both treated and untreated individuals had an HG strength test below the 50th percentile (68.8% and 78.9%, respectively). In addition, a significant difference in the number of patients having severe HG deficiency (< 5th percentile) among treated individuals (36.8%) compared to untreated (6.3%) was found (*p* = 0.047) (Figure 3D).

#### 3.4.3 Thirty‐Second Chair Stand Test

The entire group performed a mean of 60.9% (SD 15.8) of the predicted number of repetitions, which was significantly lower than the reference values [31] (Table 5). The untreated group showed a slightly higher percentage of predicted reference values than the treated group (64.7% vs. 58.0%), particularly among females.

#### 3.4.4 Isometric Muscle Strength Test

Isometric strength of the thighs was 3.8 N·m (SD 2.0) in males and 3.1 N·m (SD 1.2) in females. Corrected unilateral values were below the lowest limit of normal reference values in 61.8% of the entire cohort (60.0% in males and 62.5% in females) (Table 5). Corrected unilateral values were below the lowest limit of normal reference values in five classic form patients (71.4%), 16 late‐onset/VUS patients (55.2%), and in 13 treated patients (68.4%), with a slightly higher prevalence in treated females (72.7%).

#### 3.4.5 Isokinetic Muscle Strength Test

Isokinetic mean strength of the thighs in unilateral maximum knee torque was 118.6 N·m (SD 38.5) in males and 88.4 N·m (SD 34.2) in females and 44.3 N·m (SD 22.6) in males and 61.1 N·m (SD 21.9) in females for extensors and flexors, respectively (Table 5).

The mean corrected unilateral extension torque percentage of predicted values was 68.4% (SD 19.0) in the classic phenotype group and 67.0% (SD 24.4) in the late‐onset/VUS group, both of which were lower than the reference values.

Similarly, no statistically significant differences in reduced isokinetic strength for the extensors were observed between treated and untreated patients. Nonetheless, treated patients had a lower mean percentage of torque extension (64.7% [SD 23.0]) than predicted values.

Regarding the isokinetic strength of flexors, late‐onset/VUS, treated and untreated males tended to have a lower mean percentage of torque flexion than predicted values (61.9% [SD 22.3], 66.4% [SD 19.6], 63.7% [SD 29.7], respectively).

### 3.5 Physical Habits and Perceived Fatigue

#### 3.5.1 Global Physical Activity Questionnaire

Males reported a mean of 1178.3 MET‐min/week (SD 1184.2), while females reported a mean of 991.2 MET‐min/week (SD 1179.2), both exceeding the WHO minimum recommendation of 600 MET‐min/week (Table 1). Sedentary behaviour averaged 445.0 (SD 212.1) minutes/day in males and 430.8 (SD 214.2) min/day in females (less than the threshold of 8 h/day).

#### 3.5.2 Bell CFIDS Disability Scale

Bell Scale total score for the entire sample was 85.9 (SD 12.8), with no significant differences observed for sex and phenotype (Table 6). On the other hand, treated participants had a lower mean score (79.1 [SD 12.9]) compared to untreated participants (91.3 [SD 9.6]) (*p* = 0.02). This difference was statistically significant in males (*p* = 0.024) and females (*p* = 0.011), with treated patients consistently scoring lower.

**TABLE 6:** Fatigue Impact and Bell Scale scores in males, females and the total cohort (*N* = 37) stratified by phenotype and ERT/chaperon treatment.

Variables	All sample (*n*)		FD phenotype (*n*)					ERT/chaperon therapy (*n*)				
			Classic		Late‐onset/VUS			Treated		Untreated		
FIS40 total score	Total (37)	29.8 ± 27.0	Total (9)	46.8 ± 27.3	Total (28)	24.3 ± 24.9	***p* = 0.048** *	Total (21)	38.1 ± 28.8	Total (16)	18.1 ± 20.3	***p* = 0.022** *
	Males (12)	34.3 ± 26.6	Males (4)	45.5 ± 35.0	Males (8)	28.8 ± 22.0	*p* = 0.428	Males (9)	31.8 ± 26.8	Males (3)	42.0 ± 30.1	*p* = 0.636
	Females (25)	27.6 ± 27.4	Females (7)	47.8 ± 23.9	Females (18)	22.6 ± 26.3	*p* = 0.079	Females (12)	42.9 ± 30.5	Females (13)	13.5 ± 14.1	***p* = 0.005** *
		*p* = 0.483		*p* = 0.915		*p* = 0.267			*p* = 0.386		*p* = 0.239	
FIS40 psychological score	Total (37)	13.6 ± 14.5	Total (9)	22.8 ± 13.9	Total (28)	10.6 ± 13.6	***p* = 0.038** *	Total (21)	17.2 ± 14.9	Total (16)	8.8 ± 12.9	*p* = 0.076
	Males (12)	18.6 ± 16.5	Males (4)	23.8 ± 18.4	Males (8)	16.0 ± 16.2	*p* = 0.504	Males (9)	16.0 ± 14.5	Males (3)	26.3 ± 23.2	*p* = 0.529
	Females (25)	11.6 ± 13.1	Females (7)	22.0 ± 11.5	Females (18)	8.5 ± 12.5	*p* = 0.055	Females (12)	18.1 ± 15.8	Females (13)	4.8 ± 4.8	***p* = 0.008** *
		*p* = 0.190		*p* = 0.875		*p* = 0.261			*p* = 0.758		***p* = 0.004** *	
FIS40 physical score	Total (37)	9.8 ± 8.2	Total (9)	15.8 ± 9.6	Total (28)	7.9 ± 6.8	***p* = 0.009** *	Total (21)	13.1 ± 8.3	Total (16)	5.4 ± 5.7	***p* = 0.002** *
	Males (12)	10.2 ± 7.7	Males (4)	15.2 ± 11.4	Males (8)	7.6 ± 4.1	*p* = 0.110	Males (9)	10.6 ± 8.6	Males (3)	9.0 ± 5.6	*p* = 0.731
	Females (25)	9.6 ± 8.5	Females (7)	16.2 ± 9.4	Females (18)	8.0 ± 6.7	*p* = 0.124	Females (12)	15.0 ± 7.8	Females (13)	4.6 ± 5.6	***p* = 0.001** *
		*p* = 0.842		*p* = 0.897		*p* = 0.886			*p* = 0.243		*p* = 0.306	
FIS40 cognitive score	Total (37)	6.4 ± 7.0	Total (9)	8.2 ± 7.9	Total (28)	5. ± 6.7	*p* = 0.435	Total (21)	7.9 ± 8.2	Total (16)	4.6 ± 4.5	*p* = 0.156
	Males (12)	5.5 ± 6.6	Males (4)	6.5 ± 10.5	Males (8)	5.1 ± 4.5	*p* = 0.816	Males (9)	5.2 ± 7.2	Males (3)	6.7 ± 5.0	*p* = 0.718
	Females (25)	6.8 ± 7.2	Females (7)	9.6 ± 6.1	Females (18)	6.2 ± 7.5	*p* = 0.315	Females (12)	9.8 ± 8.6	Females (13)	4.1 ± 4.4	***p* = 0.044** *
		*p* = 0.603		*p* = 0.625		*p* = 0.659			*p* = 0.198		*p* = 0.477	
Bell Scale total score	Total (37)	84.3 ± 13.0	Total (9)	79.4 ± 12.9	Total (28)	85.9 ± 12.8	*p* = 0.212	Total (21)	79.1 ± 12.9	Total (16)	91.3 ± 9.6	***p* = 0.003** *
	Males (12)	84.2 ± 15.6	Males (4)	77.5 ± 18.9	Males (8)	87.5 ± 13.9	*p* = 0.394	Males (9)	80.0 ± 15.8	Males (3)	96.7 ± 5.8	***p* = 0.024** *
	Females (25)	84.4 ± 11.8	Females (7)	81.0 ± 7.4	Females (18)	85.3 ± 12.7	*p* = 0.352	Females (12)	78.3 ± 10.9	Females (13)	90.0 ± 10.0	***p* = 0.011** *
		*p* = 0.964		*p* = 0.746		*p* = 0.699			*p* = 0.790		*p* = 0.181	

*Note:* Data are expressed as mean ± standard deviation.

Abbreviations: FIS, Fatigue Impact Scale; SD, standard deviation; VUS, Variant of Uncertain Significance.

* Statistically significant.

#### 3.5.3 Fatigue Impact Scale (FIS‐40)

The FIS40 total score for the entire sample was 29.8 (SD 27.0) (Table 6). The classic phenotype group exhibited a higher mean score (46.8 [SD 27.3]) compared to those with the late‐onset/VUS phenotype group (24.3 [SD 24.9]) (*p* = 0.048) (Table 6). Similarly, treated participants had a higher mean score (38.1 [SD 28.8]) than untreated participants (18.1 [SD 20.3]) (*p* = 0.022), particularly among females (*p* = 0.005).

The FIS40 psychological subscore for the entire sample was 13.6 (SD 14.5). Classic phenotype group scored higher (22.8 [SD 13.9]) compared to the late‐onset/VUS phenotype group (10.6 [SD 13.6]) (*p* = 0.038). Treated females scored higher than untreated ones (*p* = 0.008).

FIS40 physical sub‐score for the total sample was 9.8 (SD 8). Classic phenotype group scored higher (15.8 [SD 9.6]) than the late‐onset/VUS phenotype group (7.9 [SD 6.8]) (*p* = 0.009). Treated patients had a higher score (13.1 [SD 8.3]) compared to untreated participants (5.4 [SD 5.7]) (*p* = 0.002), particularly among females (*p* = 0.001).

FIS40 cognitive sub‐score was 6.4 (SD 7.0), with no significant differences among sex, phenotype, or therapy, except for lower scores in untreated females (*p* = 0.044).

## 4 Discussion

This study described, for the first time, the impact of sex, phenotype and ERT/chaperone treatment on physical fitness, through cardiopulmonary function and body composition, and on physical function, evaluated by muscle performance tests and self‐reported fatigue, in FD patients.

Our findings reveal a sex‐ and phenotype‐dependent vulnerability in aerobic capacity among FD patients, with males and patients with the classic phenotype experiencing the most pronounced impairments, possibly reflecting earlier and more severe multi‐systemic involvement.

Despite their small sample sizes, these findings are consistent with previous studies on exercise capacity [12, 16, 32]. Bierer et al. identified a reduction in V̇O_2_ peak in 15 FD patients, which improved with ERT, but the underlying mechanisms were not explored [16]. Similarly, Powell et al. found diminished V̇O_2_ peak in 29 FD patients compared to healthy controls [32].

More recently, Roy et al. demonstrated significantly impaired aerobic capacity in 42 FD patients (median age, 54 years; 62% male), even in the early stages of cardiomyopathy when conventional imaging methods failed to detect abnormalities (V̇O_2_ peak: 28.7 [7.7] mL/kg/min) [33]. The authors concluded that exercise intolerance may partly be explained by early cardiac sphingolipid accumulation [34], although other potential mechanisms contributing to reduced exercise capacity were not investigated [33].

While these findings align with our data on reduced aerobic capacity, the interpretation of its underlying causes differs. Indeed, in 50% of our cases, a reduced VO_2_ on CPET was attributed to peripheral aetiology with no evidence of cardiac or ventilatory limitation, particularly among individuals with the classic phenotype. Additionally, the interplay between reduced aerobic capacity and altered body composition profiles, particularly increased fat mass and reduced muscle mass, may indicate a vicious cycle in FD patients that perpetuates functional decline [15, 18, 33].

Another key impairment observed in our cohort was pronounced muscle weakness, particularly in handgrip strength and lower‐limb function. This may reflect that peripheral muscular dysfunction, rather than cardiovascular or ventilatory limitations alone, plays a central role in the reduced physical function and exercise capacity observed in FD patients, especially those with the classic phenotype. However, the role of additional factors, such as endothelial dysfunction, immune dysregulation, and inflammation, cannot be excluded.

Among markers of inflammation, phase angle has emerged as an indirect biomarker associated with reduced muscle mass and malnutrition [35, 36, 37]. In our sample, PA in males was significantly reduced in subjects receiving ERT/chaperone treatment and positively correlated with higher VO_2_ peak predicted percentage values. These findings suggest that impaired functional capacity, resulting from altered body composition, may be attributed to increased oxidative stress and systemic inflammation [36]. A possible explanation is that the accumulation of glycosphingolipids leads to mitochondrial dysfunction, oxidative damage [38, 39] and chronic inflammation. These effects damage muscle fibers, impair energy production and disrupt cellular homeostasis. As a result, muscular capacity and exercise tolerance are reduced, along with altered energy metabolism, ultimately impairing physical performance and endurance. These effects appear to be more severe in males with the classic phenotype. Longitudinal studies are warranted to further clarify these pathophysiologic mechanisms.

In the second step of our analysis, we found that patients with the classic form reported greater fatigue than those with late‐onset/VUS, as anticipated. Unexpectedly, treated individuals also reported higher fatigue levels, suggesting that treatment status may primarily reflect underlying disease severity rather than therapeutic response. However, these associations likely result from baseline disease burden or treatment selection bias rather than direct effects of therapy.

To our knowledge, no previous study has investigated fatigue in FD patients using these two tools [17].

The study's strength lies in its assessment of physical fitness and function, providing a detailed analysis across sex, phenotype and treatment status (ERT/chaperone therapy). Notably, the classic phenotype emerged as a key factor of reduced muscular strength, exercise intolerance and impaired quality‐of‐life outcomes.

However, this study has several limitations. First, its cross‐sectional design limits the ability to establish causal relationships between clinical variables, treatment status and functional outcomes, as well as to assess the disease's long‐term impact on functional impairment. Future research should prioritize larger, longitudinal studies to validate the hypothesis that exercise intolerance in FD patients may be closely linked to consequent and mutually causative physical inactivity, rather than disease progression alone. Second, the small sample size and the lack of a control group limit the generalizability of the findings and reduce statistical power, particularly for subgroup analyses. Further studies should investigate the pathophysiological mechanisms and adjuvant treatments [40] that contribute to sex and phenotype‐related differences. Third, due to the limited sample size, we did not adjust for disease severity using multivariable modelling. However, descriptive data on phenotype classification and treatment suggest greater disease severity in the treated group, as all individuals with the classic phenotype were receiving ERT. This context should be considered when interpreting outcome comparisons, particularly given that, as frequently observed, treated patients in our sample had poorer physical fitness scores. Fourth, the physical fitness assessment included body composition measurements, cardiorespiratory endurance and muscular fitness, but not musculoskeletal flexibility [41]. However, cardiorespiratory capacity remains the most critical component of physical fitness due to its strong correlation with overall health status [42]. Fifth, handgrip strength values were referenced from a Canadian population, which may not fully correspond to the Italian context. However, to our knowledge, no standardized normative values for HG are currently available for the Italian population.

Lastly, self‐reported data on physical activity levels should be interpreted with caution, even when using validated tools. Longitudinal studies using wearable devices are necessary to capture physical activity levels more accurately [25].

In conclusion, this study demonstrates that FD patients with the classic phenotype, particularly males, are associated with lower physical fitness and functional levels. Cardiopulmonary exercise testing, bioelectrical impedance analysis, and simple functional tests (e.g., handgrip strength test and chair‐stand tests) should be integrated into the routine assessment of FD patients. Early intervention and ongoing monitoring may help mitigate exercise intolerance and physical impairments.

## Funding

Open access funding provided by Università degli Studi di Verona. This research received no external funding.

## Ethics Statement

The present study has been approved by the Research Ethics Committee of the University Hospital of Padova (Ref. No. 6125/AO/24, AOP3572) and has therefore been conducted in accordance with the ethical standards outlined in the 1964 Declaration of Helsinki and its subsequent amendments. All participants gave their informed consent prior to their inclusion in the study. Details that might disclose the identity of the subjects under study have been omitted.

## Conflicts of Interest

The authors declare no conflicts of interest.

## Supporting information


**Table S1:** Descriptions of instruments used to assess the physical and functional parameters in FD patients.
