# Supplementary material for: Physical Fitness and Physical Function in Patients With Fabry Disease: A Cross‐Sectional Multicentre Study
Source: J Cachexia Sarcopenia Muscle. 2026 Feb 18;17(1):e70233. doi: 10.1002/jcsm.70233 (PMC12916441; doi:10.1002/jcsm.70233)
Supplement: Supplementary file 1 — Table S1: Descriptions of instruments used to assess the physical and functional parameters in FD patients. [file JCSM-17-e70233-s001.docx]

# **Supplementary Table 1.** Descriptions of instruments used to assess the physical and functional parameters in FD patients.

| **Parameter** | | **Measure** | | **Method** | | **Patient condition and procedure** | | **References** | |
| --- | --- | --- | --- | --- | --- | --- | --- | --- | --- |
| BMI | | kg/m^2^ | | Mechanical scale with movable weights and an altimeter (Seca 700, weight precision: 50 g; height precision: 0.5 cm;  Seca gmbh & co kg, Hammer Steindamm 3-25, 22089 Hamburg - Germany) | | At fast for at least four hours before the measurement | | World Health Organisation (WHO) [1] | |
| Body composition | | Total body water (TBW)  Intracellular and extracellular water (ICW, ECW)  Free fat body mass (kg and %)  Free fat mass index (kg/m^2^)  Fat mass (kg and %)  Fat mass index (kg/m^2^)  Phase angle | | Multi-frequency bioelectrical impedance analysis (BIA); frequencies: 1 kHz, 5 kHz, 50 kHz, 250 kHz, 500 kHz, and 1000 kHz;  InBody S10, InBody Co., Ltd., 24 Garak-ro 16-gil, Songpa-gu, Seoul, Republic of Korea. Software: LookingInbody ®. | | Six frequencies to measure multiple parameters and segmental distribution. The device is calibrated according to the manufacturer’s guidelines prior to data collection to ensure accuracy and reliability.  Participants are prepared under standardized conditions to minimize variability.  Patients are instructed to fast for at least four hours before the test, avoid alcohol or caffeine for 24 hours, and remove any conductive materials such as jewelry.  Measurements are taken with participants in a supine position, ensuring proper placement of electrodes on the hands, feet, and other required contact points as specified by the device protocol. | | Standard values for Italian population [2,3] | |
| Non-invasive cardiopulmonary exercise test (CPET) | | Respiratory exchange ratio (RER)  Peak heart rate (HR)  Peak oxygen consumption (VO_2_ peak)  Oxygen uptake efficiency slope (OUES) | | It is performed using one of the following protocols:   - Incremental Bruce ramp protocol - Modified Bruce ramp protocol on treadmill (T170DE-med, h/p/cosmos, Nussdorf-Traunstein, Germany) - 10Wx1min to 15Wx1min ramp on cycle ergometer (eBike, GE Healthcare)   Jaeger (Vyaire Medical GmbH, Vyntus CPX, Hoechberg, Germany) | | All tests are ECG-monitored and maximal according to exhaustion criteria of Borg rating (≥18/20) associated with a RER ≥1.10 and/or a peak HR ≥85% of predicted maximal HR, and/or the achievement of a plateau of VO_2_ peak. Predicted maximal HR is calculated as: (220-age) bpm. Peripheral SpO2 and blood pressure are monitored during the whole test.  Respiratory gas exchange (VO2, VCO2) and ventilation are monitored breath-by-breath during the whole test. Ventilation/carbon dioxide production slope (VE/VCO2 slope) is calculated as the coefficient of linear regression obtained by plotting the VE and VCO2 data from the beginning of the exercise (removing possible initial hyperventilation) to the respiratory compensation point (RCP).  O_2_pulse early flattening is defined by a plateau of O_2_pulse <50% of exercise phase duration. The OUES is calculated as the coefficient of the relationship between oxygen uptake and the logarithm of total ventilation. | | American Thoracic Society standards [4]  Standard values [5] | |
| Handgrip (HG) strength test | | kg | | Calibrated hydraulic hand dynamometer (Baseline® Evaluation Instruments, Elmsford, NY, USA) | | Dominant and non-dominant grip strength from sitting position, neutral relaxed shoulder, 90° flexion of the elbow and neutral wrist. For the analysis, an average of three standardized measurements is considered.  The values are categorized into percentiles. | | Canadian standard values [6] | |
| 6-minute walk test (6MWT) | | m | | Indoor hallway, along a straight, flat, well-lit corridor; the evaluator will pay attention to safety and offer support if necessary | | Total distance is calculated at the end of the test with 1 meter sensibility, then compared to standard equation and lower limits of normal.  Patients are asked for a Visual Analogic Scale of Fatigue (VAS-F) at the end of the test and a Dyspnea Borg Scale (from a minimum shortness of breath of 0 to a maximum of 10) at the beginning and at the end of the test (15). | | American Thoracic Society guidelines [7]  Standard equation for total meters [8]  Borg Scale [9] | |
| 30-second chair stand test (30-STS) | | n. of repetitions | | Adult chair | | It involves performing as many repetitions as possible within a predetermined 30-second period of rising from and sitting down on a chair.  The starting position is seated with the upper limbs crossed over the chest, and a repetition is counted when the individual rises, completes the extension of the hip joint, and returns to the seated position. | | Standard procedure [10]  Standard values [11] | |
| Short Physical Performance Battery (SPPB) | | m/s  n. of repetitions  seconds  total score (1-12) | | Indoor hallway, Adult chair | | SPPB scale consists of 3 timed components:  1) a 4-meter usual pace walk (Gait Speed test),  2) a five-repetition chair stand without using one’s arms (Sit-and-stand Test),  3) a progressive test of standing balance (Balance Test).  Specifically, the walking component is performed by the participant two times and the best trial is used. Times from each component is scored from 0-4, with higher scores corresponding to better performance.  Finally, all three component scores is added together, and a final total SPPB scores is ranged between 4 and 12, with 4-6 representing the low, 7-9 the middle, and 10-12 the best performances based on previously established clinical cut points. | | International guidelines [12] | |
| Isometric strength of thigh extensors | | Mean peak torque (N·m) | | Isokinetic machine (bilateral knee isometric strength, bilateral knee isokinetic strength).  Isometric and isokinetic measurements are performed with an isokinetic dynamometer to assess lower limb strength.  The device is a multi-joint evaluation system (Prima Plus, Easytech, Italy), composed by a sit and a mechanic unit. | | Maximum extensors’ contraction of both thighs for five seconds, from a sitting position with blocked hip and 70° flexion of the knees (average peak torque value for two trials). | | Predicted values formula by Nejc Sarabon et al. [13], converting bilateral to unilateral strength values using mean BI (bilateral index) by Jakob Skarabon review [14] | |
| Isokinetic muscle strength of the knee, both for extensors and flexors | | Mean peak torque (N·m) | | Isokinetic machine (bilateral knee isometric strength, bilateral knee isokinetic strength).  Isometric and isokinetic measurements is performed with an isokinetic dynamometer to assess lower limb strength.  The device is a multi-joint evaluation system (Prima Plus, Easytech, Italy), composed by a sit and a mechanic unit. | | Five repetitions, from maximum flexion to maximum extension of both thighs, using an angular velocity of 60°/sec (mean value of each parameter for two trials). | | Standard values [15] | |
| Global Physical Activity Questionnaire (GPAQ) | | MET-min/week  Daily minutes of sedentary behavior | | Questionnaire | | Self-report | | Standard Values [16] | |
| Bell CFIDS disability scale | | Total score (0-100) | | Questionnaire | | Self-report | | Standard Values [17] | |
| Fatigue Impact Scale (FIS-40) | | Total score  Psychological subscore (FIS40P)  Physical subscore (FIS40F)  Cognitive subscore (FIS40C) | | Questionnaire | | Self-report | | Standard Values [18] | |

BIA, bioelectrical impedance analysis; BMI, body mass index; CFIDS, Chronic Fatigue Immune Dysfunction Syndrome; CPET, cardiopulmonary exercise test; ECW, extracellular water; FIS-40, Fatigue Impact Scale; GPAQ, Global Physical Activity Questionnaire; HR, heart rate; ICW, intracellular water; MET, metabolic equivalent of task; OUES, oxygen uptake efficiency slope; RER, respiratory exchange ratio; SpO₂, peripheral oxygen saturation; TBW, total body water; 6MWT, six-minute walk test; VAS-F, Visual Analog Scale for Fatigue; VE/VCO₂, ventilation/carbon dioxide production slope; VO₂, volume of oxygen consumption.

# **References**

1. Obesity : Preventing and Managing the Global Epidemic : Report of a WHO Consultation Available online: https://iris.who.int/handle/10665/42330 (accessed on 21 January 2025).

2. Coin, A.; Sergi, G.; Minicuci, N.; Giannini, S.; Barbiero, E.; Manzato, E.; Pedrazzoni, M.; Minisola, S.; Rossini, M.; Del Puente, A.; et al. Fat-Free Mass and Fat Mass Reference Values by Dual-Energy X-Ray Absorptiometry (DEXA) in a 20-80 Year-Old Italian Population. *Clin Nutr* **2008**, *27*, 87–94, doi:10.1016/J.CLNU.2007.10.008.

3. Anja, B.W.; Danielzik, S.; Dörhöfer, R.P.; Later, W.; Wiese, S.; Müller, M.J. Phase Angle from Bioelectrical Impedance Analysis: Population Reference Values by Age, Sex, and Body Mass Index. *JPEN J Parenter Enteral Nutr* **2006**, *30*, 309–316, doi:10.1177/0148607106030004309.

4. Weisman, I.M.; Marciniuk, D.; Martinez, F.J.; Testing Measurements Frank Sciurba, E.; Sue, D.; Myers Bruce Johnson, J.; Whipp, B.; Zeballos, J.; Beck, K.; Mahler, D.; et al. ATS/ACCP Statement on Cardiopulmonary Exercise Testing. *https://doi.org/10.1164/rccm.167.2.211* **2012**, *167*, 211–277, doi:10.1164/RCCM.167.2.211.

5. Dores, H.; Mendes, M.; Abreu, A.; Durazzo, A.; Rodrigues, C.; Vilela, E.; Cunha, G.; Gomes Pereira, J.; Bento, L.; Moreno, L.; et al. Cardiopulmonary Exercise Testing in Clinical Practice: Principles, Applications, and Basic Interpretation. *Rev Port Cardiol* **2024**, *43*, 525–536, doi:10.1016/J.REPC.2024.01.005.

6. Grip Strength Reference Values for Canadians Aged 6 to 79: Canadian Health Measures Survey, 2007 to 2013 - ARCHIVED Available online: https://www150.statcan.gc.ca/n1/pub/82-003-x/2016010/article/14665-eng.pdf (accessed on 21 January 2025).

7. Crapo, R.O.; Casaburi, R.; Coates, A.L.; Enright, P.L.; MacIntyre, N.R.; McKay, R.T.; Johnson, D.; Wanger, J.S.; Zeballos, R.J.; Bittner, V.; et al. ATS Statement: Guidelines for the Six-Minute Walk Test. *Am J Respir Crit Care Med* **2002**, *166*, 111–117, doi:10.1164/AJRCCM.166.1.AT1102.

8. Cazzoletti, L.; Zanolin, M.E.; Dorelli, G.; Ferrari, P.; Dalle Carbonare, L.G.; Crisafulli, E.; Alemayohu, M.A.; Olivieri, M.; Verlato, G.; Ferrari, M. Six-Minute Walk Distance in Healthy Subjects: Reference Standards from a General Population Sample. *Respir Res* **2022**, *23*, doi:10.1186/S12931-022-02003-Y.

9. Lee, K.A.; Hicks, G.; Nino-Murcia, G. Validity and Reliability of a Scale to Assess Fatigue. *Psychiatry Res* **1991**, *36*, 291–298, doi:10.1016/0165-1781(91)90027-M.

10. Jones, C.J.; Rikli, R.E.; Beam, W.C. A 30-s Chair-Stand Test as a Measure of Lower Body Strength in Community-Residing Older Adults. *Res Q Exerc Sport* **1999**, *70*, 113–119, doi:10.1080/02701367.1999.10608028.

11. McKay, M.J.; Baldwin, J.N.; Ferreira, P.; Simic, M.; Vanicek, N.; Burns, J.; Nightingale, E.; Pourkazemi, F.; Sman, A.; Hiller, C.; et al. Reference Values for Developing Responsive Functional Outcome Measures across the Lifespan. *Neurology* **2017**, *88*, 1512–1519, doi:10.1212/WNL.0000000000003847.

12. Welch, S.A.; Ward, R.E.; Beauchamp, M.K.; Leveille, S.G.; Travison, T.; Bean, J.F. The Short Physical Performance Battery (SPPB): A Quick and Useful Tool for Fall Risk Stratification Among Older Primary Care Patients. *J Am Med Dir Assoc* **2021**, *22*, 1646–1651, doi:10.1016/J.JAMDA.2020.09.038.

13. Šarabon, N.; Kozinc, Ž.; Perman, M. Establishing Reference Values for Isometric Knee Extension and Flexion Strength. *Front Physiol* **2021**, *12*, doi:10.3389/FPHYS.2021.767941.

14. Škarabot, J.; Cronin, N.; Strojnik, V.; Avela, J. Bilateral Deficit in Maximal Force Production. *Eur J Appl Physiol* **2016**, *116*, 2057–2084, doi:10.1007/S00421-016-3458-Z.

15. Zhang, Y.; Chen, K.; Liu, K.; Wang, Q.; Ma, Y.; Pang, B.; Huang, L.; Ma, Y. New Prediction Equations for Knee Isokinetic Strength in Young and Middle-Aged Non-Athletes. *BMC Public Health* **2023**, *23*, doi:10.1186/S12889-023-17478-7.

16. Bull, F.C.; Maslin, T.S.; Armstrong, T. Global Physical Activity Questionnaire (GPAQ): Nine Country Reliability and Validity Study. *J Phys Act Health* **2009**, *6*, 790–804, doi:10.1123/JPAH.6.6.790.

17. OI Resource - CFIDS Disability Scale Available online: https://www.oiresource.com/cfsscale.htm (accessed on 21 January 2025).

18. Piscitelli, D.; Brichetto, G.; Geri, T.; Battista, S.; Testa, M.; Monti Bragadin, M.; Pellicciari, L. Italian Adaptation and Psychometric Validation of the Fatigue Impact Scale (FIS) and Its Modified Versions in Adults with Multiple Sclerosis: A Rasch Analysis Study. *Disabil Rehabil* **2024**, doi:10.1080/09638288.2024.2302878.
